# Pulmonary Thromboembolism in a Patient With Behçet’s Disease: A Case Report

**DOI:** 10.1155/carm/8323943

**Published:** 2026-04-20

**Authors:** Elham Kalantari, Mohammad Emami Ardestani

**Affiliations:** ^1^ Division of Pulmonary and Critical Care Medicine, Department of Internal Medicine, School of Medicine, Isfahan University of Medical Science, Isfahan, Iran, mui.ac.ir

**Keywords:** Behçet’s disease (BD), deep vein thrombosis (DVT), pulmonary thromboembolism (PTE), vasculitis

## Abstract

Behçet’s disease (BD) is a rare, chronic, and multisystemic vasculitis characterized by recurrent oral and genital ulcers, uveitis, and skin lesions. Although vascular involvement is common in BD, pulmonary manifestations are rare and potentially fatal. Among these, pulmonary thromboembolism (PTE) represents an exceptional event, as deep vein thrombosis (DVT) in BD usually remains adherent to the vessel wall and seldom embolizes to the lungs. We report a 52‐year‐old male with BD who presented with a four‐day history of progressive dyspnea, productive cough, and new‐onset hemoptysis. He had been in clinical remission for one year after discontinuing colchicine therapy. On admission, his oxygen saturation was 90% on room air, with inspiratory crackles on auscultation. Laboratory findings revealed leukocytosis, elevated inflammatory markers, and a markedly increased D‐dimer level. CT pulmonary angiography demonstrated multiple filling defects in the right main and segmental pulmonary arteries without evidence of pulmonary artery aneurysm (PAA). Anticoagulation with heparin was initiated. However, during hospitalization, the patient developed oral and genital aphthous ulcers, indicating disease relapse. Immunosuppressive therapy with cyclophosphamide and prednisolone led to significant clinical improvement. This case underscores the importance of maintaining a high index of suspicion for PTE in BD and highlights the necessity of distinguishing BD‐related thrombosis from conventional embolic events, as management requires a combination of immunosuppressive and anticoagulant therapy to prevent fatal complications.

## 1. Introduction

Behçet’s disease (BD) is a rare, chronic, and systemic inflammatory disorder clinically characterized by a triad of symptoms, including recurrent oral and genital aphthae, ocular inflammation, and skin lesions. BD can affect a wide range of organs in the body, particularly blood vessels of all sizes. In fact, vascular involvement is one of the most prominent manifestations of this disease, with vascular inflammation (vasculitis) and deep vein thrombosis (DVT) being the most frequently observed vascular complications [1]. DVT, a serious medical condition characterized by blood clots forming in deep veins, primarily in the lower extremities, is a significant risk factor for pulmonary thromboembolism (PTE). However, despite a high prevalence of DVT in BD, it rarely leads to PTE. That is likely due to the strong adherence of blood clots within inflamed veins [2].

In BD, other pulmonary complications are also relatively rare but result in serious medical conditions. When the lungs are affected by this disease, the most frequent clinical manifestation is the formation of pulmonary artery aneurysms (PAAs), which are often associated with hemoptysis [3]. Therefore, early diagnosis and treatment of BD are crucial for managing its symptoms and preventing complications, especially when the lungs and blood vessels are involved. Here, we present a rare case of PTE in a patient with BD. This case is particularly interesting because PTE is an uncommon manifestation of BD. Reporting such a case adds valuable information to the limited literature on pulmonary vascular complications of BD without PAA formation.

## 2. Case Presentation

A 52‐year‐old male with a medical history of BD, who had been previously diagnosed based on recurrent oral and genital ulcers, lower limb arthralgia, and arthritis, presented to the hospital following a four‐day history of progressively worsening dyspnea and a productive cough. On the day of admission, the patient reported the onset of hemoptysis. Additionally, he experienced pleuritic chest pain in the right posterolateral region, along with dyspnea on exertion. The patient had a history of taking colchicine for the management of BD, which was discontinued one year prior without any recurrence of symptoms.

At admission, his vital signs were as follows: temperature of 38°C, respiratory rate (RR) of 25 per minute, O_2_ saturation of 90% on room air, blood pressure of 120/80 mmHg, and pulse rate of 84 per minute. During the physical examination, the patient exhibited mild respiratory distress. An inspiratory crackle was detected at the lung bases. Moreover, there were no signs of an aphthous ulcer in the oral and genital mucus membrane, nor was there clinical evidence of DVT.

To address the patient’s respiratory symptoms, meropenem, a broad‐spectrum antibiotic, was initiated. Diagnostic tests, including blood tests and CT pulmonary angiogram (CTPA), were then performed to identify the underlying cause of the patient’s symptoms and to differentiate whether these symptoms were associated with infection or attributable to BD.

The blood tests revealed leukocytosis (14000/µL), with a differential count showing neutrophilia (75.2%) and lymphopenia (16.6%). In addition, hemoglobin of 11.4 g/dL indicated mild anemia. Laboratory investigations demonstrated elevated inflammatory markers, including C‐reactive protein (CRP) of 62 mg/L and erythrocyte sedimentation rate (ESR) of 94 mm/hr. The D‐dimer level was remarkably elevated at 1937 ng/mL fibrinogen equivalent unit (FEU). Notably, the platelet counts were in the normal range (289,000). Furthermore, autoimmune markers, including antinuclear antibody (ANA) and anti‐neutrophil cytoplasmic antibody (ANCA), were negative (Table 1).

**TABLE 1:** Laboratory findings at admission.

Parameter	Result	Reference range	Unit
White blood cell (WBC) count	14,000	4000–10,000	/μL
Neutrophils	75.2	40–70	%
Lymphocytes	16.6	20–45	%
Hemoglobin (Hb)	11.4	13–17	g/dL
Platelet count	289,000	150,000–400,000	/μL
Erythrocyte sedimentation rate (ESR)	94	< 20	mm/hr
C‐reactive protein (CRP)	62	< 10	mg/L
D‐dimer	1937	< 500	ng/mL FEU
Antinuclear antibody (ANA)	Negative	Negative	—
Anti‐neutrophil cytoplasmic antibody (ANCA)	Negative	Negative	—
Blood urea nitrogen (BUN)	21	7–23	mg/dL
Serum creatinine	0.9	0.6–1.3	mg/dL
Alanine aminotransferase (ALT)	23	10–49	U/L
Aspartate aminotransferase (AST)	25	10–40	U/L
Total bilirubin	0.7	0.2–1.2	mg/dL
Prothrombin time (PT)	13.2	11–14	sec
Activated partial thromboplastin time (aPTT)	34	25–35	sec
International normalized ratio (INR)	1.0	0.9–1.2	—

*Note:* Table 1 summarizes the laboratory parameters obtained upon admission. The results revealed leukocytosis, neutrophilia, elevated inflammatory markers (CRP and ESR), and markedly increased D‐dimer levels, consistent with systemic inflammation and thrombotic activity. Autoimmune markers (ANA and ANCA) were negative.

A thrombophilia workup was not pursued during the acute phase, as the thrombotic event occurred in the context of active BD relapse, which is a well‐established prothrombotic inflammatory state. The patient had no history of smoking, recent immobilization, long‐haul travel, malignancy, or hormonal therapy. Screening for secondary causes of thrombosis, including renal and hepatic dysfunction, was unremarkable. HLA‐B∗51 testing was not performed, as it would not have altered acute management or long‐term therapeutic decisions.

**FIGURE 1:**
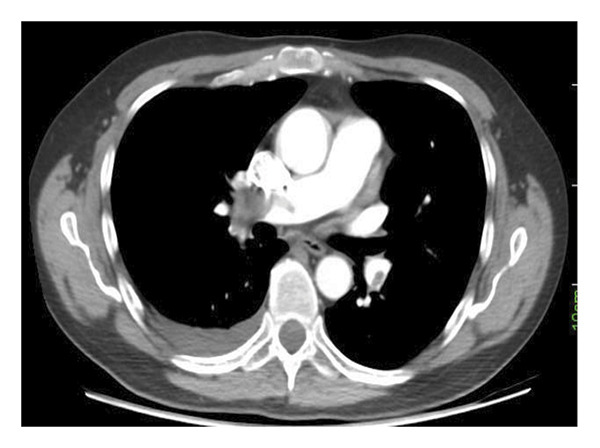
Filling defect in right main pulmonary artery and left lower lobe pulmonary artery.

**FIGURE 2:**
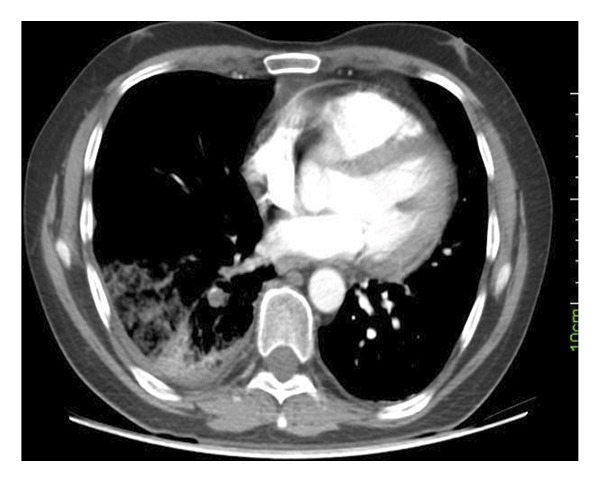
Axial CT image showing wedge‐shaped peripheral consolidation in the right lower lobe, suggestive of pulmonary infarction. Filling defect in the right lower lobe pulmonary artery is clearly visible.

The CTPA findings, as presented in Figures 1 and 2, indicated the presence of segmental and subsegmental wedge‐shaped alveolar opacities in the right lower lobe, which were consistent with hemorrhage and infarction. Furthermore, filling defects within the right main artery, extending to the right lobar and segmental branches, were revealed, suggesting PTE. No evidence of PAAs was observed on the CTPA. Following these findings, a heparin infusion was started.

A rheumatology consultation was requested. Given that the patient had been clinically quiescent for BD over the preceding year and was not receiving ongoing therapy and in the absence of PAA on CTPA, the initial impression was that the PTE might be unrelated to active BD. However, during hospitalization, the patient developed recurrent oral and genital aphthous ulcers, strongly suggesting disease relapse with vascular involvement. Consequently, immunosuppressive therapy with cyclophosphamide and systemic corticosteroids was initiated, in addition to anticoagulation. Following clinical stabilization, the patient was discharged on oral prednisolone and warfarin.

Immunosuppressive treatment consisted of monthly intravenous cyclophosphamide administered according to standard vasculitis protocols (750 mg/m^2^ per cycle) for a total of 12 cycles, combined with oral prednisolone initiated at 1 mg/kg/day. Corticosteroid therapy was gradually tapered over six months based on clinical and laboratory response. After completion of cyclophosphamide induction therapy, azathioprine (2 mg/kg/day) was introduced as maintenance treatment and continued for approximately one year.

The patient was followed for five years after the initial hospitalization. During follow‐up, dyspnea and hemoptysis resolved completely, and no recurrent thromboembolic or respiratory events were observed. Corticosteroids were successfully discontinued, followed by withdrawal of azathioprine after sustained clinical remission. At the last follow‐up visit, the patient remained clinically stable and was maintained on colchicine 1 mg daily, reporting only occasional minor oral aphthous ulcers without other systemic manifestations of BD.

## 3. Discussion

BD is a chronic, complex, and multisystemic inflammatory disorder that is considered a systemic vasculitis with diverse manifestations, including vascular involvement. Although DVT is a relatively common vascular complication in patients with BD, it rarely progresses to PTE. This may be attributed to the strong adherence of blood clots within inflamed veins, which potentially limits the embolization of thrombi into the pulmonary circulation [2]. Pulmonary involvement in BD is infrequent. However, it has been documented that when it occurs, the first and most common clinical manifestation is PAA. Other possible involvements are pulmonary hemorrhage, pulmonary infarction, PTE, arteriovenous shunt in the lung, and aneurysmal fistula [3].

The results of a large‐scale cohort study involving 6075 Iranian adult patients with BD revealed that 9.1% experienced vascular involvement, with DVT being the most prevalent at 6.6%. In contrast, the pulmonary involvement rate was notably less than 1%, precisely 0.97%. PTE accounted for just 0.18% of the cohort [4].

This case describes the rare but potentially life‐threatening complication of BD, PTE, in a 52‐year‐old man who had been previously diagnosed with BD. On presentation day, his chief complaint was dyspnea, hemoptysis, and pleuritic chest pain.

PAA and PTE may present with similar symptoms, including cough, hemoptysis, and dyspnea; however, their management strategies differ significantly. The primary therapeutic approach for PAA typically includes immunosuppressive medications to address the inflammation of the vessel wall. In contrast, while anticoagulation is the standard treatment for PTE, it can increase the risk of rupture in PAA. Consequently, accurate differential diagnosis between PAAs and PTE is essential for optimizing patient outcomes. Another challenge in managing BD with pulmonary involvement is distinguishing between PTE and in situ thrombosis, as there is no diagnostic gold standard and their clinical manifestations often overlap. It has been suggested that in situ thrombosis typically affects peripheral pulmonary arteries. Key feature on CTPA that helps differentiate in situ thrombosis from PTE includes filling defects. In situ thrombosis typically presents as eccentric filling defects that are more adherent to the vessel wall, whereas PTE usually appears as rounded, free‐floating emboli [5].

Besides, in situ thrombosis can be distinguished from chronic thromboembolic pulmonary hypertension (CTEPH) by the absence of mismatched perfusion defects on the ventilation/perfusion (V/Q) scan because in situ thrombosis is nonobstructive [6].

In our case, the CTPA findings indicated the presence of PTE and no evidence of PAA. Based on these findings, heparin therapy was started. Despite the initial diagnosis that PTE might be unrelated to BD due to the patient’s recent symptom‐free period and the absence of PAAs, the subsequent recurrence of oral and genital ulcers strongly supported the diagnosis of BD‐related PTE.

Although the patient had been clinically stable for approximately one year prior to presentation, the development of PTE may reflect ongoing subclinical vascular inflammation, which is increasingly recognized in BD even during apparent remission. Previous studies have shown that vascular endothelial dysfunction and prothrombotic immune activation can persist despite the absence of overt mucocutaneous or systemic symptoms. Discontinuation of colchicine, which has known anti‐inflammatory and antithrombotic effects, may have contributed to loss of vascular protection in this patient. Furthermore, relapse of BD became clinically evident during hospitalization with the recurrence of oral and genital aphthous ulcers, supporting the notion that thrombotic events may precede or herald clinical reactivation of the disease. These observations emphasize that thromboembolic complications in BD can occur independently of apparent disease activity and highlight the importance of maintaining vigilance for vascular events even during periods of clinical remission.

Given the absence of PAA on CTPA, anticoagulation was initiated in combination with immunosuppression, with rapid clinical improvement. Current management of refractory vascular BD increasingly includes biologic agents such as anti–tumor necrosis factor (TNF) or anti–interleukin‐6 therapies. However, in this case, conventional immunosuppressive therapy with cyclophosphamide and corticosteroids was selected due to the acute presentation, availability, and the presence of extensive vascular involvement, resulting in excellent clinical response and long‐term stability [7].

This case aligns with previous reports [8–12] that describe PTE as a rare but significant complication of BD. The current report, along with the earlier ones, highlights the importance of a high index of suspicion for vascular events in patients with BD, regardless of their current disease activity status (active or in remission), and emphasizes the critical need for early diagnosis and intervention in patients with BD, particularly important in those presenting with respiratory symptoms.

## Funding

No funding was received for this manuscript.

## Consent

No written consent has been obtained from the patient as there are no patient identifiable data included in this case report.

## Conflicts of Interest

The authors declare no conflicts of interest.

## Data Availability

Data supporting the findings are available from the corresponding author upon reasonable request.
